# 

**DOI:** 10.6026/973206300211843

**Published:** 2025-07-31

**Authors:** Kaveeta Ramesh Kumar, Nora Sharafli, Ramesh Kumar

**Affiliations:** 1 Department of Obstetrician and Gynaecologist, Zulekha Hospital, Sharjah, UAE

**Keywords:** Non-invasive prenatal testing (NIPT), cell-free fetal DNA, global implementation, prenatal screening, ethical issues, healthcare integration, aneuploidy, policy, AI in healthcare

## Abstract

Non-invasive prenatal testing (NIPT) offers a highly sensitive and specific method for screening fetal chromosomal abnormalities using cell-free fetal DNA from maternal plasma. This systematic review examined global implementation strategies, highlighting clinical, ethical and policy-related barriers and facilitators. NIPT is well-integrated in high-resource settings, while cost and regulatory issues limit access in lower-income countries. Ethical implementation requires informed consent, equitable access and continuous policy evaluation. Findings provide key recommendations for global healthcare systems integrating NIPT.

## Background:

Prenatal testing is essential to obstetric care and provides an early opportunity to identify chromosomal anomalies and genetic disorders in the developing fetus. Historically, prenatal diagnostic testing has relied on invasive amniocentesis and chorionic villus sampling (CVS), which provided accurate results, but came at some level of risk (*e.g.*, risk of miscarriage) to the mother and/or the fetus as well as maternal discomfort [1]. These invasive procedures were meant for high-risk pregnancies and not everyone used them as a screening option [1]. As the demands for means of testing that were earlier, safer and more accessible began to emerge; advances in molecular genetics and analysis of fetal DNA would usher in the means of testing fetus non-invasively. These methods would ultimately change and significantly improve prenatal care and afford wider population screening with little if no risk to either the mother and/or fetus [2]. Non-invasive prenatal testing (NIPT) represents the evolution of prenatal screening that offers a safe and validated alternative to traditional invasive diagnosis [3]. As a clinical implementation, NIPT was introduced in 2011, as it measures the fetal - cell-free DNA (cffDNA) in maternal plasma to screen for diseases caused by common chromosomal aneuploidies, such as Down syndrome (trisomy 21), or trisomy 18 (Williams syndrome), trisomy 13 (Patau syndrome). NIPT has reported 99% sensitivity for some disease (Wang *et al.* 2021) [3]. NIPT is safe for both low and high-risk pregnancies as it can be offered and performed at the same time as other non-invasive testing, whereas amniocentesis and chorionic villus sampling procedures, which can be performed later in pregnancy, can lead to miscarriages among 0.2% and 2% of cases respectively [4]. MPS (massively parallel sequencing) technology has led to dramatic gains in NIPT sensitivity and specificity [4]. Over the last ten years, NIPT has evolved beyond aneuploidy screening to include a screen for sex chromosome abnormalities, microdeletions and genome-wide analysis which has led to ongoing debate surrounding NIPTs clinical utilization, ethical implications and incorporation into established public health systems globally [5].

NIPT has rapidly been adopted worldwide but there is notable inconsistency in how it is being implemented from country to country and health system to health system. Consequently, there are differences in the access to and nature of clinical practice and differences in regulation [5]. NIPT has been shown to be superior in terms of accuracy and safety to traditional invasive methods, however, despite its recommended clinical application, issues including cost, ethical considerations and the barrier of integrating into routine prenatal care need to be addressed to help standardize its use [6]. Additionally, increased application of NIPT and new technologies related to NIPT - hybrid- developments - will require ongoing evaluation of its clinical use and policy considerations. Given the diversity of prenatal screening in different countries across the world and its evolving nature as technology continues to progress, the need for a systematic review is warranted to summarize the available evidence on international implementation of NIPT, illustrate barriers and effective practices. The review will provide a broad overview of NIPT implementations to help inform policy, clinicians and researchers moving forward as well as assist with developing an evidence-based and standardized way to practice NIPT globally [7]. The main objective of this systematic review is to analyze how non-invasive prenatal testing (NIPT) has been implemented in different parts of the world and the main issues related to its introduction in different health care services. Specifically, the review will aim to (1) examine how NIPT has been adopted in high-, mid- and low-income countries; (2) look at the clinical, ethical, economic and social reasons for using NIPT; (3) consider differences in terms of access, awareness and regulation; and (4) highlight opportunities for equitable and ethical implementation. Certainly! Here's a refined version of your sentence while keeping the first part unchanged [8]. Therefore, it is of interest to offer informed recommendations that hold significance for health policy, guide clinical practice, and shape future research in the area of prenatal screening.

## Material and Methods:

## Search methods:

In order to identify relevant studies relating to international implementation and challenges in NIPT, a substantial literature search was conducted. The electronic databases were searched included PubMed, Scopus, Web of Science, Embase and the Cochrane Library. The search was limited to articles published in English from January 2011 to March 2025, reflecting the time that has elapsed since NIPT was first introduced clinically. A combination of Medical Subject Headings (MeSH) and free-text terms were used in the search. The primary terms included: ("non-invasive prenatal testing" OR "NIPT" OR "cell-free fetal DNA" OR "cfDNA") AND ("implementation" OR "adoption" OR "integration") AND ("challenges" OR "barriers" OR "ethical issues" OR "cost-effectiveness") AND ("global" OR "international" or country/region names). The search was equally refined by using Boolean operators (AND, OR). Additionally resources were identified by manually reviewing the reference lists of key studies and review articles. Grey literature such as government reports and policy briefs that were appropriate to obtain larger implementation were also considered. All identified studies were collated into a reference management tool (*e.g.*, EndNote, Mendeley) for de-duplication and screening for relevance.

## Inclusion and exclusion criteria:

Review included studies relevant to non-invasive prenatal testing (NIPT), particularly research that discussed clinical implementation, accessibility, integration into healthcare systems and challenges associated with implementation. Included studies involved pregnant individuals or healthcare providers and provided data from any country in order to encompass a global sense. Both quantitative and qualitative original research articles, systematic reviews and policy reports were to be included. Studies published from January 2011 up to March 2025 (the period following clinical introduction of NIPT) were to be included in the review, irrespective of the publication status, except items needed for the systematic but registered prospective NIPT study. Studies were excluded if they were considered non-clinical (i.e., considered lab studies if no clinical applicability). Editorials, opinions, or conference abstracts were excluded unless they provided enough data related to the review objectives. Articles that only discussed traditional invasive prenatal testing methods, or otherwise did not deal with NIPT, were excluded. We excluded studies that did not have available full text articles and studies that were flagged as duplicates.

## Data extraction and synthesis:

Data extraction was conducted systematically with a standardized form created to extract relevant study characteristics and outcomes pertaining to the review objectives. The extracted data included authorship, year of publication, country or region of study, study design, population characteristics and implementation focus for NIPT, announced challenges for NIPT, any mentioned outcomes and recommendations. Two independent reviewers conducted the data extraction to ensure accuracy and reliability and discrepancies were resolved through discussion or with a third reviewer. The extracted data were then arranged thematically and qualitatively synthesized that allowed for the identification of common patterns, differences by region and themes emerging across studies. Where applicable, we organized the findings based on income nation classification and healthcare systems to allow for comparative thoughts on implementation and barriers globally. Figure 1 (see PDF) illustrates the PRISMA flowchart depicting the selection process of the included article.

## Quality assessment of included studies:

To ensure the findings were reliable and valid, the quality of all studies was formally assessed. The type of study guided which critical appraisal tools were used. The Critical Appraisal Skills Programme (CASP) checklist was used for qualitative studies. For quantitative observational studies, the PRISMA (Preferred Reporting Items for Systematic reviews and Meta-Analyses) guidelines were used. For mixed-method studies, criteria from both tools were used. Each study was independently assessed by two reviewers on methodological rigor, clarity of research aims, appropriateness of study design, sampling strategy, method of data collection and transparency on reporting findings. Any disagreements were settled by discussion or consultation with a third reviewer. No studies were excluded due exclusively to quality scores, but quality assessment informed the interpretation of the findings and overall strength of conclusions drawn from the synthesis. Table 1 (see PDF) shows the overview of non-invasive prenatal testing.

## Overview of NIPT technology:

Invasive prenatal testing (NIPT) relies on the analysis of cell-free fetal DNA (cffDNA) in maternal blood. CffDNA primarily comes from placental trophoblasts and it can be identified as early as the 5th week of gestation, although it is typically most accurate after the 10th week. The principle behind NIPT lies in isolating and analyzing the cffDNA using different genomic technologies, with massively parallel sequencing (MPS) being the most widely used among them. MPS allows researchers to quantify the cffDNA fragments that correspond to specific chromosomes and, ultimately, suggests chromosomal aneuploidies (such as trisomy 21, 18 and 13) using high sensitivity and specificity. Other approaches that are mentioned in the literature include digital PCR and microarray analyses, but they are less often used to conduct NIPT in practice. Compared to traditional invasive testing, (*e.g.*, amniocentesis and chorionic villus sample), NIPT is attractive because it is the first non-invasive approach with some of the following benefits: safety and reduced risk of miscarriage and the ability to test relatively early for fetal aneuploidies. Results also need to be interpreted with some of the potential technical limitations in mind (*e.g.*, maternal DNA interference, low fetal fraction, or falsely identified aneuploidies with scenarios, such as confined placental mosaicism) that may result in false negatives and false positives [6, 7, 8, 9, 10-11]. NIPT is primarily used to assess common fetal chromosomal aneuploidies; the most commonly analyzed conditions are trisomy 21 (Down syndrome), trisomy 18 (Edwards syndrome) and trisomy 13 (Patau syndrome) and are correlated with the greatest developmental and health-related issues and are also some of the most common chromosomal anomalies identified in early pregnancy. In addition to the above trisomy conditions, many advanced NIPT platforms now examine additional sex chromosome aneuploidies, such as Turner syndrome (45,X), Klinefelter syndrome (47,XXY), Triple X (47,XXX) and XYY syndrome (47,XYY) and in some cases also include detection of microdeletion syndromes (*e.g.*, 22q11.2 deletion syndrome (DiGeorge syndrome). The performance characteristics, including accuracy and clinical utility, are variable for these smaller chromosomal deletions. More recently, broadening NIPTs panels have become available and can also target the detection of rare autosomal aneuploidies, alongside copy number variants (CNVs) or kinds of variants, now using more advanced techniques such as whole genome sequencing (WGS) and even single gene disorders. The clinical utility, validation and ethical concerns related to this broader spectrum of conditions remains an area of on-going research) [12, 13- 14].

Non-invasive prenatal testing (NIPT) has proven to have excellent accuracy and reliability when screening for common chromosomal aneuploidies. NIPT has over 99% sensitivity and around 99.9% specificity for trisomy 21 (Down syndrome), which makes it the most accurate non-invasive clinical screening currently available. The NIPT has slightly lower sensitivities and specificities for trisomy 18 and trisomy 13, but remain high with sensitivity between 91-97% and specificity above 99%. The accuracy of NIPT relies significantly on the fetal fraction, the percentage of cell-free fetal DNA in the maternal blood, which typically needs to be above 4% in order for NIPT to yield reliable results. Fetal fraction can vary according to many factors, including maternal weight, gestational age and placental health, all of which can impact the NIPT reliability. Despite the low false-positive rate of NIPT when compared to the traditional serum screening, false-positive and false-negative results may occur from confined placental mosaicism, vanishing twin syndrome and maternal chromosomal mutations. Therefore, while NIPT is highly reputable as a screening test, it is NOT diagnostic. Any positive NIPT result must be confirmed via an invasive diagnostic test such as amniocentesis or chorionic villus sampling (CVS) [15, 16, 17, 18, 19-20].

There is substantial disparity in the global implementation of Non-Invasive Prenatal Testing (NIPT) that is attributable to differences in economic, infrastructure/availability, cultural and policy factors. An overview of NIPT implementation in high-income countries, middle- and low-income countries, as well as comparative implementation strategies and incorporation within health care systems follows, with supporting literature. High-income countries (HICs) have rapidly adopted and integrated NIPT into prenatal care. Netherlands, the United Kingdom and some areas of the United States represent HICs that have integrated NIPT as part of their national screening program and fractional or second-tier testing after initial risk assessments. Countries with NIPT integrated into the health care system have seen considerable reductions in invasive diagnostic testing and complications as a result of this testing. Importantly, inequities exist within HICs, whereby socioeconomically disadvantaged groups exhibit lower rates of uptake of NIPT. For instance, in the Netherlands, NIPT uptake was 20.3% in socio-economically disadvantaged neighbourhoods compared to 47.6% in other neighbourhoods [11, 21 and 22]. In lower- and middle-income countries (LMICs), NIPT faces barriers to implementation including costs, infrastructure and lack of trained personnel. For example, in India, NIPT was brought into limited markets for accessibility and affordability reasons. Most governments in low- and middle-income countries cannot afford to subsidize NIPT, so it becomes inaccessible for most members of their population. If NIPT can be implemented in LMICs, equitable access will require investment in sequencing capacity and training for public health workers [5, 10].

## Comparative implementation strategies:

Two main and global strategies of NIPT implementation have emerged as follows:

[1] First-tier screening: Some countries provide NIPT as a first-tier screening test for all pregnant women from which to obtain diagnostic information after early detection of aneuploidy. This is potentially beneficial for early identification, but NIPT may be costly [21].

[2] Second-tier screening: Other countries use NIPT as second-tier screening test to be used in women considered high-risk following a first-tier test. This is notionally more cost-effective. However, this could delay aneuploidy diagnosis [23]. Each implementation strategy has strengths and weaknesses, with implementation often contingent on a country's clinical settings, geography and resources.

## Integration of healthcare systems:

Attention must be given to the ethical, legal and social implications of integrating NIPT into healthcare systems. In Canada, the project decision-makers acknowledged the need for responsible implementation, ensuring that reasonable accommodations for informed consent and equitable access to healthcare could be met. The process of integration in Australia specifically identified challenges in enacting policies that standardize service delivery and access [24]. Invasive prenatal testing (NIPT) provides safer and more accurate methods for prenatal care and the identification of chromosomal abnormalities. However, the integration into clinical practice involves a number of clinical and ethical considerations. Below is an overview of some of the key considerations:

NIPT acts as a non-invasive screening tool that identifies common chromosomal conditions, such as Down syndrome (trisomy 21), trisomy 18 and trisomy 13, through the sampling and analysis of cell-free fetal DNA in maternal blood. It is the high sensitivity and specificity of NIPT that has made it a valuable tool in prenatal screening. As a screening tool, positive NIPT results must be confirmed by diagnostic options including chorionic villus sampling (CVS) and amniocentesis in order to provide a diagnosis. Moreover, it is essential that the expectant parent receives counseling both before and after the procedure, to address the utility, benefits and limitations of the NIPT test and support them as they make decisions, therapy and reduce anxiety following test results. The accessibility and non-invasive nature of NIPT threatens to render the test routine and erode informed consent in the process. Informed and autonomous decision- making for patients must involve thorough counseling that considers the purpose of the test, the implications of the possible results and what choices may follow. A structured and stepwise approach will allow the patient to better understand and continue to make decisions aligned with their values. The approach explored the patient's values, layered information about the test and continued support for the interpretation of results and any potential next steps, if needed [25]. The dissemination of NIPT raises ethical issues, particularly concerning disability in society and if NIPT increases pressure for patients to terminate pregnancies by finding and/ or disclosing the personal information. There is the possibility NIPT could lead to less acceptance of people with disabilities and fewer ways of support for them in society. Ethics suggests NIPT should be available within a context of respecting reproductive autonomy, ensuring equity of access and counselling to limit the risk of harm [26, 27 and 28].

## Barriers and challenges:

The prohibitively high cost of NIPT is arguably the main barrier to its implementation. In Australia's public healthcare system, NIPT costs up to $500. NIPT is not subsidized or included on the Medical Benefits Schedule, meaning that many socioeconomically disadvantaged families cannot afford it. Several of the health professionals said the cost deters them from offering the test and patients from choosing it, even when the benefits are clinical. With NIPT in Lebanon and Quebec costing about USD 800, women or families must pay for if out of pocket. There are ethical considerations when we could offer the test to people who, by circumstance, can afford it [13]. Disparities in NIPT uptake from women occur, especially with socioeconomically disadvantaged people. For example, a study in the Netherlands showed a much lower uptake of NIPT for disadvantaged neighbourhoods at 20.3% in contrast to 47.6% for all other neighbourhoods. In the United States, NIPT access is also influenced by insurance. Women with public insurance actually had higher odds of receiving NIPT compared to those with private insurance. The insurance type influences the availability of tests, which ultimately impacts uptake [22, 29].

Sociocultural factors which include religious belief systems, cultural norms and varying levels of health literacy can influence acceptance and uptake of NIPT. Some communities may resist prenatal testing for ethical/moral reasons which leads to lower attendance rates. Also, a lack of awareness or understanding of NIPT's merits and limitations may limit prospective parents' ability to make informed decisions [11]. Rapid advancements in NIPT technology have outstripped the creation of comprehensive regulation in many countries. This can lead to inconsistency of test quality, counselling and ethical guidance. For example, in the Netherlands the country faced hurdles before NIPT could be integrated into the national healthcare system until new policies and guidelines to support safe and effective implementation [11].

## Opportunities and facilitators:

Recent improvements in genomics and sequencing technology have greatly improved the NIPT experience. We now have ultra-fast molecular counting and sequencing technology with single base pair resolution, allowing for the clinical delivery of NIPT for monogenic conditions such as sickle cell disease, cystic fibrosis, hemoglobinopathies and spinal muscular atrophy. Unlike many other carrier-screening tests, single-gene NIPT does not rely on paternal DNA samples, which can decrease testing sensitivity due to limited uptake. Long-read sequencing technology has also allowed researchers to study long cell-free DNA molecules, enabling detection and monitoring of pregnancy-associated conditions like preeclampsia. Altogether, these technological enhancements have broadened the potential use of NIPT [30].

Integrating new tests into existing public health systems has assisted in the best rollout of NIPT in many jurisdictions. For example, in the Netherlands, NIPT has been included as part of the national prenatal screening program, but, importantly, a complete counseling session is offered to all pregnant women prior to testing. Using this approach creates an opportunity to ensure that women have full information and that there is equal access to the NIPT. Italy is planning nationally to include NIPT into their, publicly funded, healthcare system with an acknowledgement of its potential to improve prenatal care. These examples demonstrate that systems can be put in place to effectively include NIPT as part of a public health model, while recognizing that there are associated benefits [31].

Awareness and education programs provide an important foundation for implementing NIPT. In the Netherlands, the national rollout of NIPT required a three-part mandatory blended learning program for the counselors (midwives, sonographers and obstetricians) who would be rolling the testing out. The primary goal was to increase the counselors' knowledge about prenatal aneuploidy screening and improve their attitudes towards NIPT at their professional level. Post-program, most of the counselors showed significant increases in knowledge and had positive perspectives concerning NIPT being a first-tier test. Such educational programs are important for ensuring that health care professionals are well prepared to guide patients in the decision making processes on whether to have NIPT [32, 33-34].

## Future directions:

## Research gaps:

Even with significant progress, NIPT faces varying degrees of research gaps. One prominent gap is the lack of high sensitivity for detecting certain chromosomal abnormalities and monogenic disorders. There is also a need for larger studies assessing the psychological impact NIPT results have on expectant parents and the long-term outcomes of pregnancies after NIPT (and HTS) screening. Closing these research gaps will require studies with large, diverse populations in order to be clinically useful and robust the findings.

## Potential for broader screening:

NIPT capabilities are being expanded further to cover not only common aneuploidies but also a broader spectrum of genetic conditions. The development of next generation sequencing has enabled the detection of microdeletions, duplications and single-gene disorders for a more extensive genetic assessment of the fetus. The potential to offer broader screening allows for earlier and improved diagnosis and management of a variety of conditions so that expectant parents may determine the best path forward.

## The role of AI and digital health tools:

Artificial Intelligence (AI) and digital health tools are increasingly playing a role in improving NIPT. AI algorithms can enhance accuracy in interpretation of results due to their capability to categized complex genomic data to find patterns that may be missing in more traditional strategies. Further, AI driven tool can combine NIPT results with an analyses of ultrasound findings and other clinical data to provide a narrative on risk assessment. Digital platforms can also better connect providers to patients with better communications regarding timely insightful information concerning NIPT results.

## Conclusion:

Non-Invasive Prenatal Testing (NIPT) is a highly accurate and low-risk prenatal screening tool, increasingly used in high-income countries but limited in lower-income regions due to cost and infrastructure. Its widespread adoption requires attention to policy, ethics, regulation and public education to ensure equitable access. With proper governance and integration of digital health tools like AI, NIPT can significantly enhance maternal-foetal healthcare globally.
